# Divergent evolutionary paces among eudicot plants revealed by simultaneously duplicated genes produced billions of years ago

**DOI:** 10.3389/fpls.2025.1518981

**Published:** 2025-02-18

**Authors:** Yao Wang, Jiangli Wang, Yingjie Li, Yongchao Jin, Xiyin Wang

**Affiliations:** ^1^ College of Mathematics and Science, North China University of Science and Technology, Tangshan, China; ^2^ Key Laboratory of Data Science and Application of Hebei Province, Tangshan, China; ^3^ School of Public Health and Protective Medicine, North China University of Science and Technology, Tangshan, China; ^4^ School of Life Science, North China University of Science and Technology, Tangshan, China

**Keywords:** Ks distribution, whole genome duplication, pathway enrichment analysis, Chi-square test, time correction

## Abstract

Polyploidization often occurs more than once along an evolutionary lineage to form extant plants. Major core eudicot plants share a whole-genome triplication (ceWGT), through which thousands of simultaneously duplicated genes are retained in extant genomes, providing a valuable starting line to check the difference in their evolutionary paces. Here, by characterizing the synonymous nucleotide substitutions (Ks) between these duplicates from 28 representative plants from 21 families, we checked the various evolutionary rates among plants among plants subjected to different rounds of extra polyploidization events. We found up to 68.04% difference in evolutionary rates among the selected plants. A statistical correlation analysis (correlation coefficient =0.57, at significant level = 0.01) indicated that plants affected by extra polyploidies have evolved faster than plants without such extra polyploidies showing that (additional) polyploidization has resulted in elevated genetic diversity. Comparing the plants affected by additional polyploidization and plants without it, the duplicated genes produced by the ceWGT and retained in extant genomes have gathered 4.75% more nucleotide substitutions in the former plants. By identifying the fast- and slowly evolving genes, we showed that genes evolving at divergent rates were often related to different evolutionary paths. By performing correction to evolutionary rates using a genome-scale approach, we revised the estimated timing of key evolutionary events. The present effort exploited the simultaneously duplicated genes produced by the shared polyploidization and help deepen the understanding of the role of polyploidization, especially its long-term effect in plant evolution and biological innovation.

## Introduction

Polyploidies have doubled or tripled genomes, and polyploidization increases genetic diversity and adaptability of organisms playing a crucial role in the evolutionary process of plants (Bowers et al., 2003; Paterson et al., 2004; Jiao et al., 2011; Paterson et al., 2012; Barker et al., 2016). In the last 20 years, genome sequencing efforts disclosed evidence that all plants or their ancestors have undergone genome duplication during the evolution (Wang et al., 2011a; Li et al., 2016; Murat et al., 2017; Van De Peer et al., 2017; Wang et al., 2020a) contributing to the origination, fast divergence, and establishment of new plant groups (Bowers et al., 2003; Soltis and Soltis, 2016).

Studies showed that the common ancestor of core eudicots experienced a hexaploidization or whole-genome triplication (ceWGT), approximately 130 million years ago (Jaillon et al., 2007). The ceWGT has been repeatedly confirmed in genome structure analysis of hundreds of species from different plant families, including *Arabidopsis*, apple, and poplar (Tuskan et al., 2006; Liu et al., 2017; Wang et al., 2019). After the ceWGT, many core eudicot plants and plant lineages have been affected by extra polyploidization. For example, an analysis of the cotton genome revealed that the *Gossypium* genus underwent a whole-genome quintuplication (Wang et al., 2016), the *Arabidopsis* genome showed two whole-genome duplication (WGD) shared by all Brassicaceae plants (Kaul et al., 2000; Vision et al., 2000; Barker et al., 2009; Jiao et al., 2012), and the soybean genome revealed a WGD event experienced by all leguminous plants and one specific to itself (Schmutz et al., 2010).

Duplicated genes provide one of the key sources of genetic innovation (Fang et al., 2023; You et al., 2023; Hu et al., 2024). Polyploidization produces thousands of simultaneously duplicated genes overnight. Even after the post-polypoidy genome turmoil, featured with large-scale chromosomal rearrangements, extensive gene losses, and wide-spread DNA mutations, hundreds of duplicated genes may be retained to present-day genomes. These duplicated genes are the materials to trigger and establish novel genetic functions, such as regulation pathways, and they are also the reasons to revolve standing functions and/or rewire established pathways (Xiao and Li, 2017; Clark et al., 2019; Wang et al., 2024). Owing to genome instability after polyploidization, these duplicated genes are often subjected to elevated genetic variation. The elevation in genetic variation should be caused by the buffering effect with the existence of duplicated copies, thereby contributing to the genetic novelty (Madlung, 2013; Van De Peer et al., 2017; Cheng et al., 2018). Actually, after polyploidization, especially at the early stages of neo-polyploidies, enormous genomic changes occurred, such as gene rearrangements, gene losses, and/or point mutations. Therefore, duplicated genes might have been subjected to a fast divergence and neo- and/or sub-functionalization process (Otto and Whitton, 2000; Soltis et al., 2015). Evidence showed that illegitimate recombination between homeologous chromosomes could have played an innegligible role (Gaeta and Chris Pires, 2010; Shen et al., 2021). Notably, the existence of polyploid-produced duplicated genes could contribute to genetic innovation for millions of years (Doyle and Coate, 2019). For example, in grasses, like rice and sorghum, illegitimate recombination proved ongoing between duplicated genes produced by a genome doubling that occurred approximately 100 millions of years ago (Wang et al., 2011b). Two pairs of functional genes, relating to C4 genes, were found in the affected homeologous chromosomal regions in grasses (Wang et al., 2009). A general belief is that the synonymous nucleotide substitutions, compared to nonsynonymous ones, are not much influenced by natural selection for not changing amino acids. Therefore, the distribution of synonymous nucleotide substitutions at synonymous substitution sites (Ks) is often used as a basis for determining and dating polyploidization or speciation events that have occurred in the history of a species (Vanneste et al., 2013) and using the Ks peak as an indicator to measure the rate of evolution.

Simultaneously duplicated genes by the same polyploidization suggested diverged evolutionary paces of plants derived from the polyploid ancestor. Duplicated genes produced by the grass-common whole-genome duplication (gcWGD) showed that different grasses have accumulated divergent levels of nucleotide substitutions since the gcWGD or their splits. Compared to rice, wheat, foxtail millet, sorghum, maize, and Brachypodium have evolved 4.6%–18.2% faster, while barley evolved even faster (28.1%–33.3%) (Wang et al., 2015). Notably, cucurbits evolve at considerably divergent rates. As to the paralogous genes produced by the cucurbitaceae-common whole-genome duplication (ccWGD), having occurred ~96 millions of years ago, watermelon and cucumber have evolved much faster (23.6%–27.4%) than melon. According to paralogs produced by the ceWGT, the melon species have evolved prominently faster (29.5%, 57.1%, and 59.0%) than grapevine, respectively (Wang et al., 2018). Here, grapevine is taken as a model plant to understand the genome structure and evolution of the other eudicot plants in that its genome revealed the ceWGT and retained the key features of the ancestral genome structure of the eudicot ancestor (Jaillon et al., 2007). As to the paralogs from Apiaceae plants, affected by recursive polyploidization events, celery and carrot have evolved 14.3% and 27.0% faster, respectively, than coriander, also showing much more divergent evolutionary rates among Apiaceae plants (Song et al., 2021a).

The study of evolutionary rates among plants is of biological and evolutionary importance in that it relates to the genetic innovation and evolutionary changes of genes, especially those duplicated genes, which are often the sources for establishing novel functions. It may also contribute to the understanding how new species, even new plant groups, such as genera or families, form and evolve under different ecological conditions. Though divergent evolutionary rates have been characterized in a few plant groups (Jaillon et al., 2007; Wang et al., 2018; Song et al., 2021a), a study with plants across different plant families has been unavailable. Moreover, though some pointed out that polyploidization is an evolutionary drive force, it has been still unclear whether divergent evolutionary rates among plants could be related to the number of polyploidization events. At the same time, there is a lack of in-depth understanding of whether the differences in gene evolutionary rates could be related to their biological functions or expression patterns in specific tissues. Here, we checked the evolutionary rates among 28 eudicot plants from 28 families, which share the ceWGT, explored the potential links between the evolutionary rates and polyploidization events, and assessed whether the rate variations could be related to the potential functions of genes. The present study may provide new insights into evolutionary divergence and help deepen the understanding of the role of polyploidization, especially its long-term effect in plant evolution and biological innovation.

## Results

### Inference of simultaneously duplicated genes due to ceWGT

**Figure 1:**
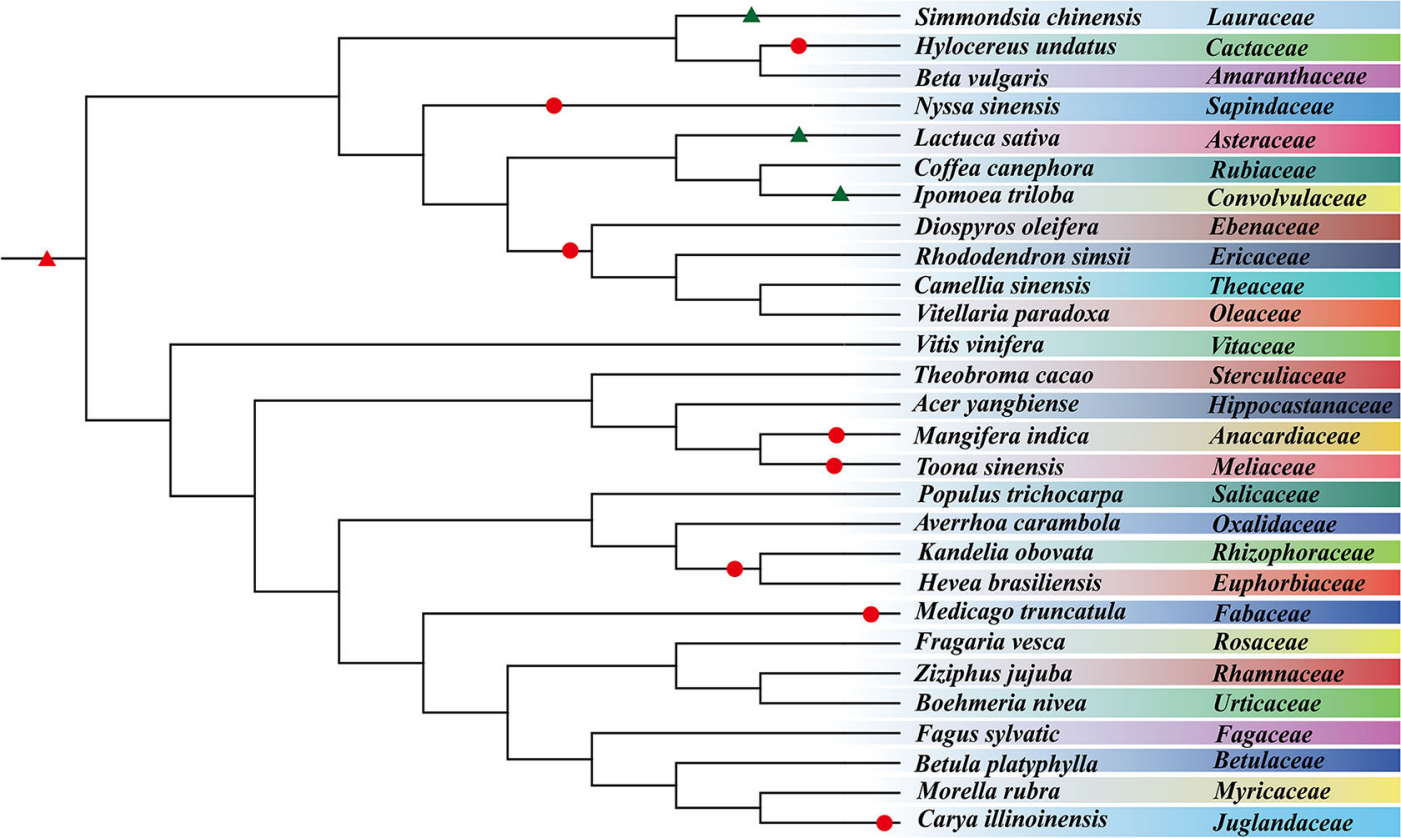
Plant phylogenetic tree. The tree involves 28 species from 14 plant orders and 28 families. Circles represent WGD events, and black triangles denote WGT events, and the ceWGT is denoted by a red triangle.

We inferred simultaneously duplicated genes produced by the ceWGT in 28 representative plants selected from 28 plant families and constructed a phylogenetic tree by integrating prior information on species relationships (**Figure 1**). First, using the most updated gene collinearity inference toolkit WGDI (Sun et al., 2022), we extracted the collinear gene pairs within each genome of the studied species. In these plant genomes, we detected 479–5,024 homologous/paralogous blocks containing 5,081–51,349 paralogous genes in collinear positions along the compared chromosomes (**Supplementary Table S2**). The mean number of duplicated blocks is 1,844, and the mean number of duplicated genes is 22,466. Among these plants, *Boehmeria nivea* has the fewest colinear blocks (479) and collinear genes (5,081), while *Hevea brasiliensis* has the highest number of collinear blocks (5,024) with 51,349 collinear genes. More collinear blocks show more chromosomal rearrangement or larger-scale DNA fractionation suggesting divergent stability among considered genomes.

**Figure 2:**
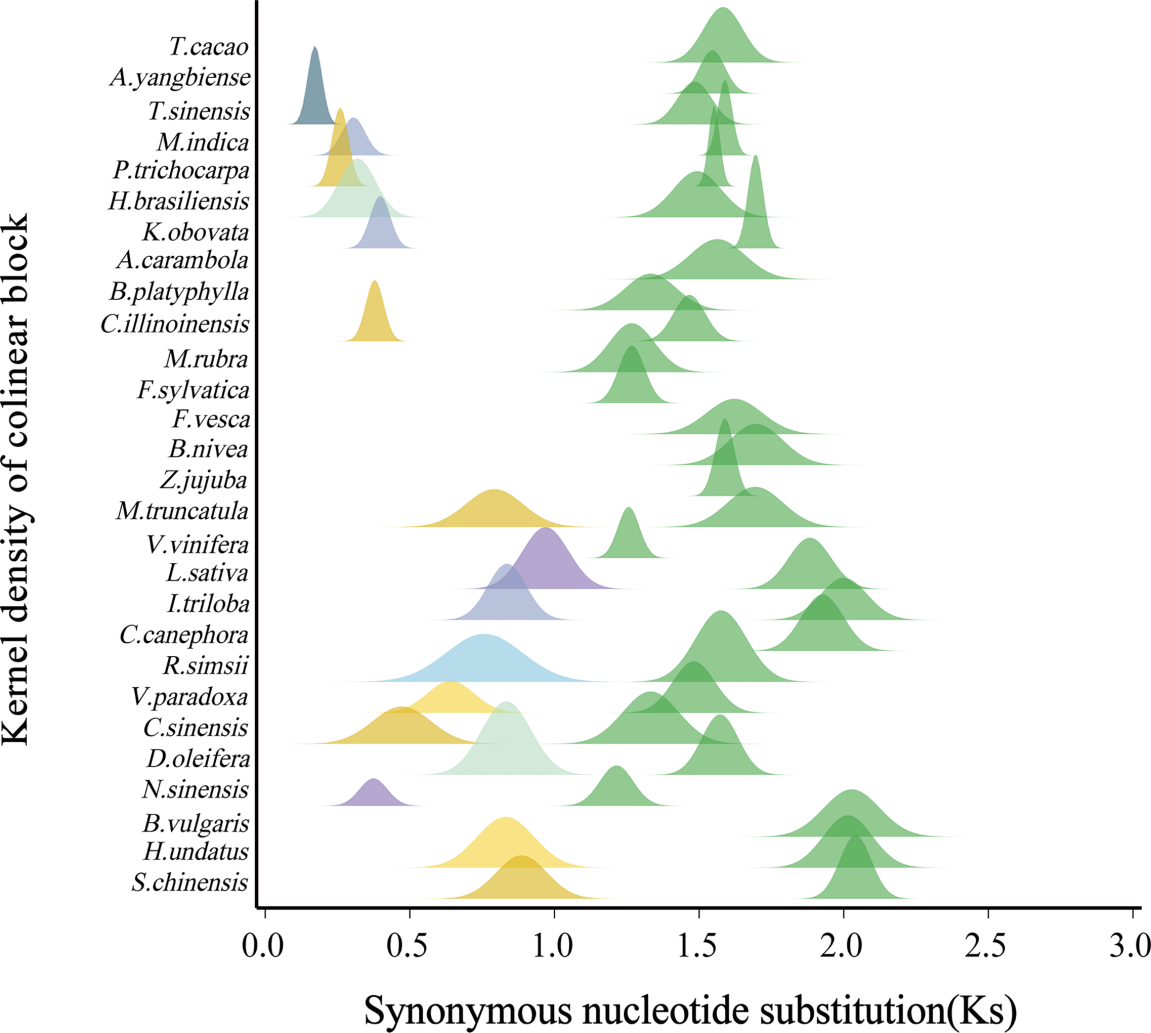
Distribution of Ks between collinearity genes from each plant genome. Fitted curves (shown in peaks) of Ks distributions in each plant. The green peaks denote the ceWGT.

**Figure 3:**
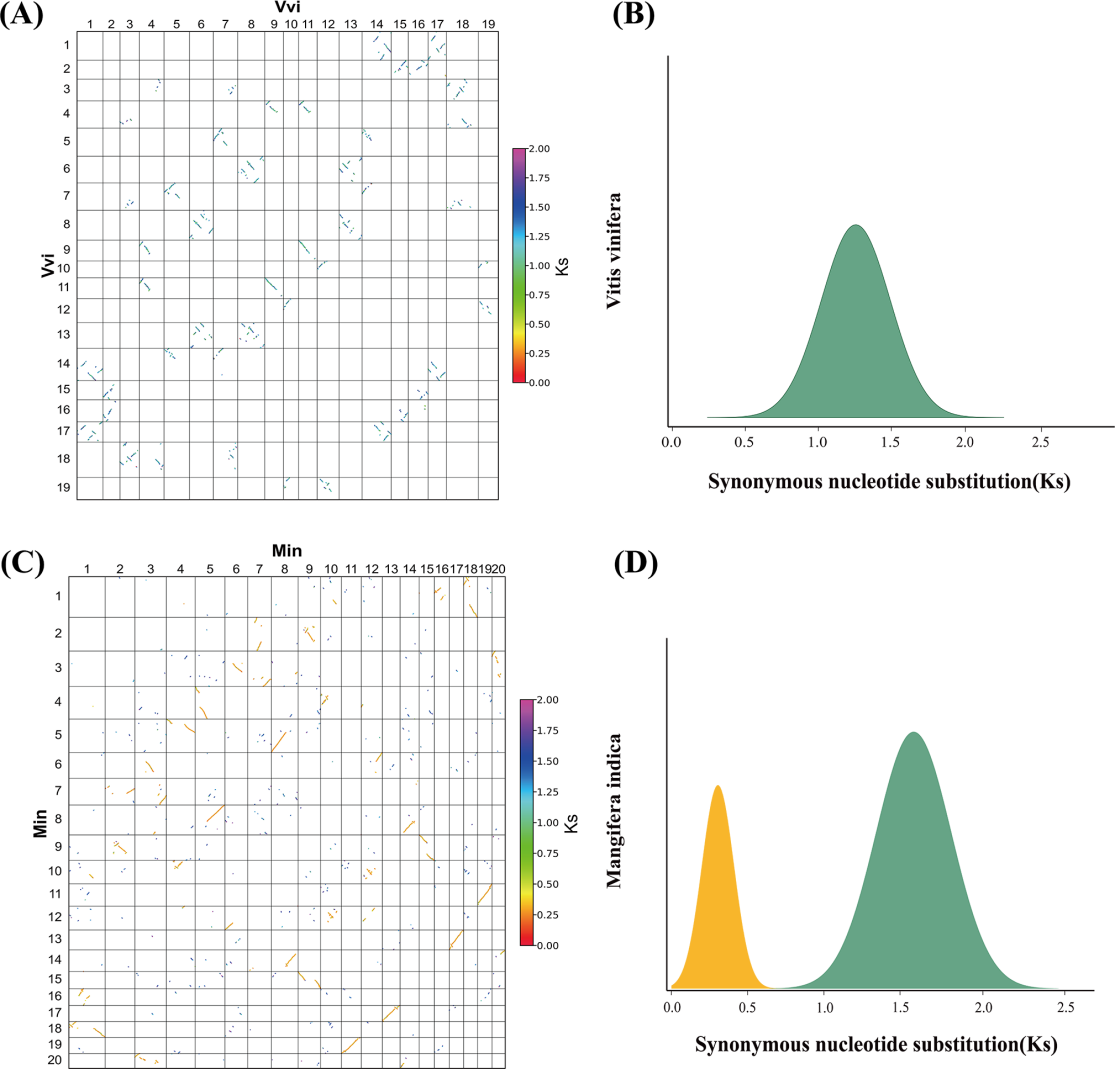
Examples of Ks distribution. **(A)** Collinear gene blocks of the *Vitis vinifera* genome. **(B)** Distribution of Ks among collinear genes from *V. vinifera*. **(C)** Collinear gene blocks of the *Mangifera indica* genome. **(D)** Distribution of Ks among collinear genes from *M. indica*.

Second, we estimated the synonymous nucleotide substitutions (Ks) between duplicates genes. This effort confirmed that each considered plant has been affected by another polyploidization event after the ceWGT and showed that the latter polyploidization often occurred tens of millions of years after the ceWGT (**Figure 2**). Considering difficulties to separate paralogous genes produced by more than two polyploidization events or by two events close in time, this study prioritizes species that each have experienced the ceWGT and an additional WGD that occurred much later than the former (**Figures 2**, **3**). This strategy facilitates a precise discrimination of paralogous genes produced by the considered polyploidization events and estimation of Ks values corresponding to each event. Besides, a check of homologous gene dot plots helped separate the ceWGT-derived duplicates from those produced by additional polyploidization (**Figure 3**). For instance, in addition to the shared ceWGT, initially revealed in grapevine, mango has undergone an additional WGD event.

Actually, the ceWGT event resulted in the production of 284–4,142 paralogous gene blocks, and 1,746–41,113 paralogous genes in these plant genomes. The most paralogous genes were found in *Fagus sylvatica*, the fewest in *Lactuca sativa*, while the average is 9,903.

Owing to long tails often present in the Ks distributions, we extracted the median Ks value of each inferred collinear gene blocks, which often provide statistically stable inference, and then characterized the Ks distributions of the medians from all collinear blocks from each genome. The obtained Ks distributions were then fitted according to the normal distribution to extract the peak value of Ks (**Supplementary Table S3**).

### Divergent evolutionary rates inferred among plants

**Figure 4:**
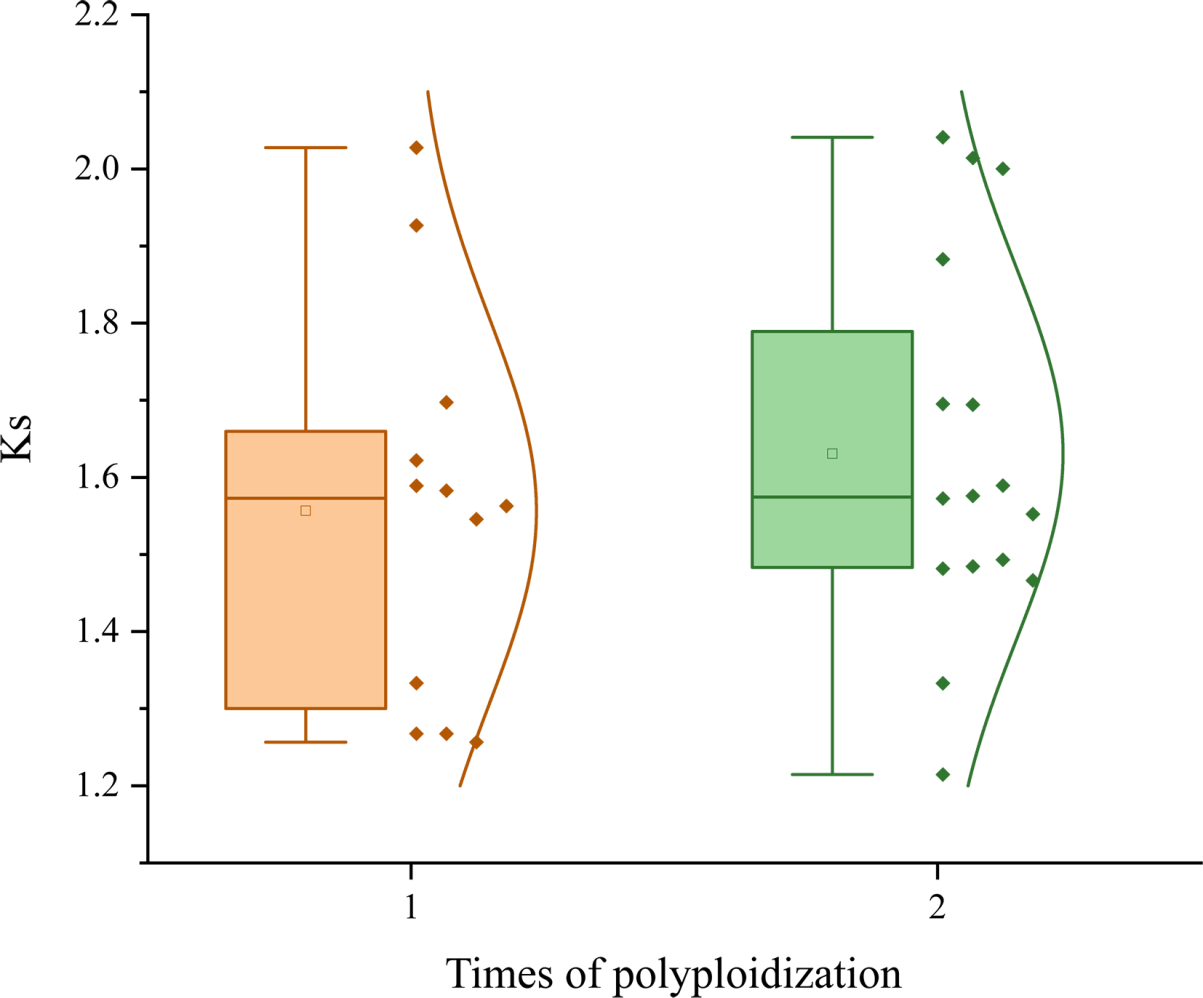
Ks distributions and rounds of polyploidization. A boxplot accompanied by a normal distribution curve illustrates the characteristics of Ks values in species affected by different numbers of polyploidization events. Yellow represents the distribution of species affected by a single polyploidization (ceWGT) event, green indicates the Ks affected by an additional polyploidization.

In our analysis, we observed divergent Ks distribution patterns among the plants under consideration. Specifically, for the shared ceWGT event, the maximum Ks value was detected in *Simmondsia chinensis*, reaching up to 2.0408, whereas the minimum was found in *Nyssa sinensis*, as low as 1.2145. The average Ks value across the shared ceWGT events was 1.5989. In contrast, for pitaya (*Hylocereus undatus*), the Ks distribution exhibited a peak at 2.0142, which is approximately two-thirds larger than the peak observed in *Nyssa sinensis*. We divided the studied plants into two groups: one group having been affected by a single round of polyploidization (the ceWGT) and the other one by additional round of polyploidization (a WGT or a WGD), which were denoted as P1R and P2R, respectively (**Figure 4**). The P1R group involves 12 plants, and the P2R includes 16 ones. For the P1R plants (including wild strawberry, *Acer yangbiense*, starfruit, cocoa tree, and grapevine), the grapevine Ks values peaked at 1.2564, much smaller than that in strawberry (Ks = 1.6221, 29.1%) and that in jujube (Ks = 1.5890, 26.5%). For species in the P2R group, an even more significant variation was observed in their Ks distribution. For instance, the ceWGT Ks value in dragon fruit peaked at 2.0142, being 51.13% higher than that in tea tree (**Figure 2**). A divergent Ks distribution shows a rather divergent evolutionary rate difference among plants, to be further discussed below.

### Additional polyploidization elevates evolutionary rates

The above characterization of the ceWGT revealed thousands of simultaneously duplicated genes in each species, which enables us to check whether these paralogous genes have gathered divergent levels of synonymous nucleotide substitutions, to further show whether the studied plants have evolved at different paces.

To explore whether polyploidization contributes to the elevation of plant evolutionary rates, we compared the two groups to find whether there is difference between them. The coefficient of variation is 0.161 for the P1R group, and 0.149 for P2R, indicating a higher degree of Ks dispersion in the former. This reflects a greater heterogeneity in the evolutionary rates among P1R species. The P1R group has a much lower average Ks (1.557 ± 0.251) than that (1.631 ± 0.243) of the P2R group (*t*-test p-value = 0.001) showing an elevated average evolutionary rate of the latter. This shows that the P2R paralogous genes produced by the ceWGT have gathered 4.75% more nucleotide substitutions than those of the P1R. Grossly, combining the above findings, no matter what the plants are or where they are originated, we showed that an additional polyploidization can significantly increase the evolutionary rates of plants. Moreover, this justify that the occurrence of polyploidization elevates a species’ evolutionary pace and results in evolutionary effects in thousands of millions of years.

### Correction to evolutionary rates facilitates uniform evolutionary dating

Divergent evolutionary paces make it difficult to perform a reasonable dating of the evolutionary events, such as ancient polyploidization or speciation of plants, under the traditional evolutionary models. These models often based their inference on the same or similar evolutionary rate(s) and other parameters. In fact, if we directly infer the occurrence time of the ceWGT, using the Ks distribution from different plants and assuming the same evolutionary rates (often a Ks rate 7.0 × 10^−9^ synonymous substitutions per site per year), we would have much diverged estimation in different plants, varying from 90 to 164 millions of years. Besides, they often used the same gene families, which evolve at paces affected by the plants from which they are derived, and divergent plant evolutionary paces were not well considered. Additionally, scientists often did not have knowledge whether the previously used (duplicated) genes had the same origination, let along whether they were produced simultaneously.

**Figure 5:**
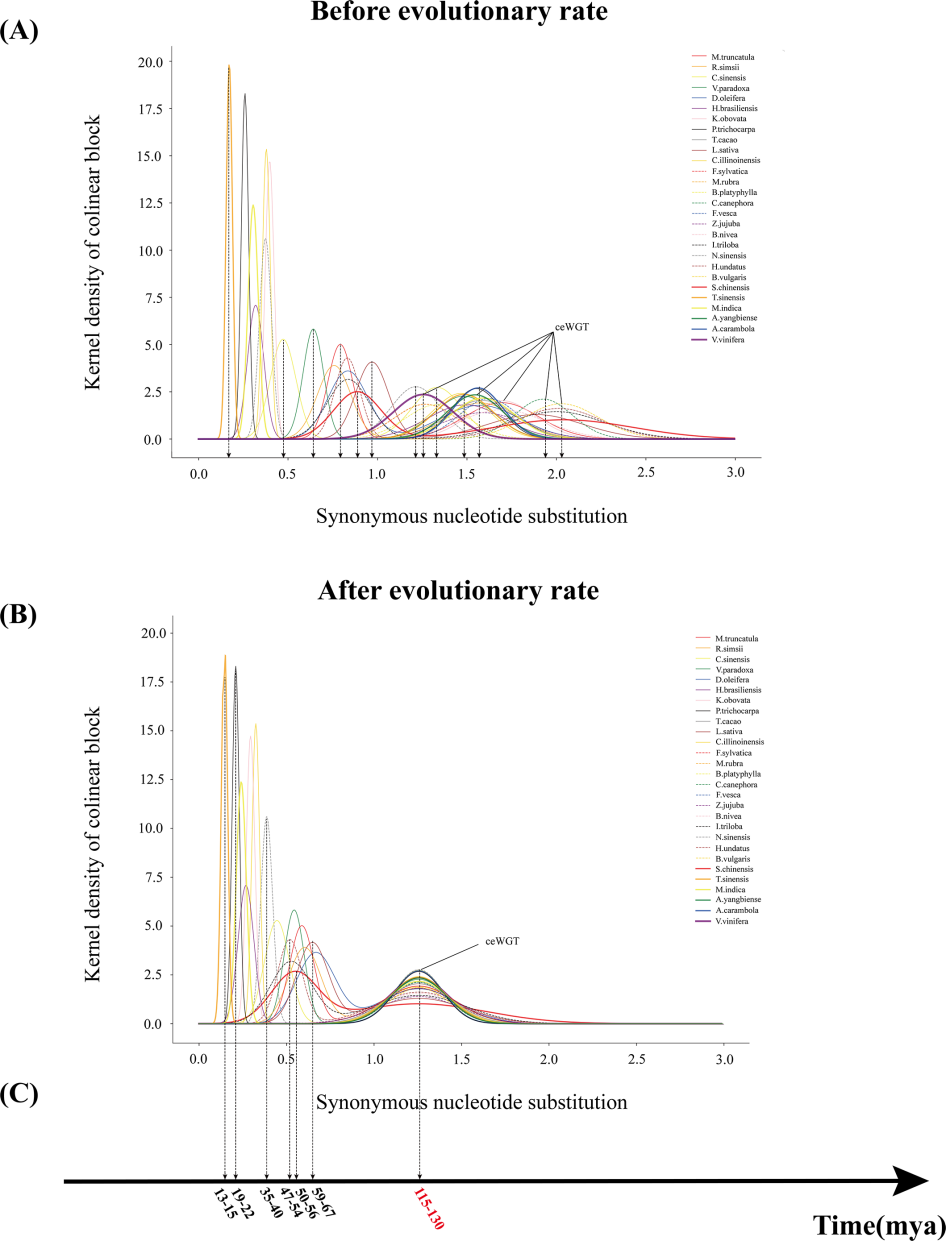
Dating evolutionary events. **(A)** Distribution of average synonymous substitutions between syntenic gene pairs in intergenomic blocks. **(B)** The corrected distribution of average synonymous substitutions, with Ks distribution curves generated using the R language, and identification of peaks and troughs. **(C)** Considering the timing of the ceWGT event to be 115–130 million years, we re-estimated the occurrence of other key evolutionary events.

Here, using genome-scale data (thousands of paralogs from each plant, simultaneously duplicated by the ceWGT), we managed to perform a correction to the Ks between paralogs from the studied plants. Grapevine was used as a reference, in that it preserved much of the genome structure of the ancestral core eudicot genome, showing a stable genome sequence during more than 100 million years of evolution. The correction was made by a linear transformation of the mean Ks values of the paralogs from the other plants to that in grapevine (see Methods for details; **Figure 5**; **Supplementary Table S4**). Actually, the Ks between the grapevine paralogs has a mean value of 1.2564, which is among the smallest ones in all plants, again showing the stable nature of the grapevine genome. After performing the correction, all of the Ks distribution peaks from different species were aligned to the same location as that of grapevine’s (**Figure 5B**).

Supposing that the ceWGT having occurred approximately 115 to 130 million years ago (Yang et al., 2020b), as previously inferred, during the Paleogene period (approximately 23 to 66 million years ago), there was a significant scale of collective polyploidization events. In this period, species, such as *M. indica*, *H. brasiliensis*, *C. illinoinensis*, and *N. sinensis*, and their respective relatives, experienced WGDs.

After correcting for the evolutionary rates, we re-estimated the timing of the most recent polyploidization events in a range of plant species (**Figure 5C**; **Supplementary Table S5**). For example, the results indicate that the recent polyploidization event in lettuce (*Lactuca sativa*) was estimated to occurred at approximately 59 to 67 million years ago (Mya), while pitaya (*Hylocereus undatus*) did so around 47 to 54 Mya. In addition, mango (*Mangifera indica*) had a specific polyploidization event occurring ~22 to 25 Mya.

### KEGG pathway enrichment analysis

Utilizing a stratification method based on the synonymous substitution rate (Ks), we systematically ranked the paralogous genes according to their Ks values in each of the selected species from 14 different orders. Considering the genes that fall within the top and bottom 20% of the Ks distribution, we identified the fast- and slowly evolving genes in each species.

**Figure 6:**
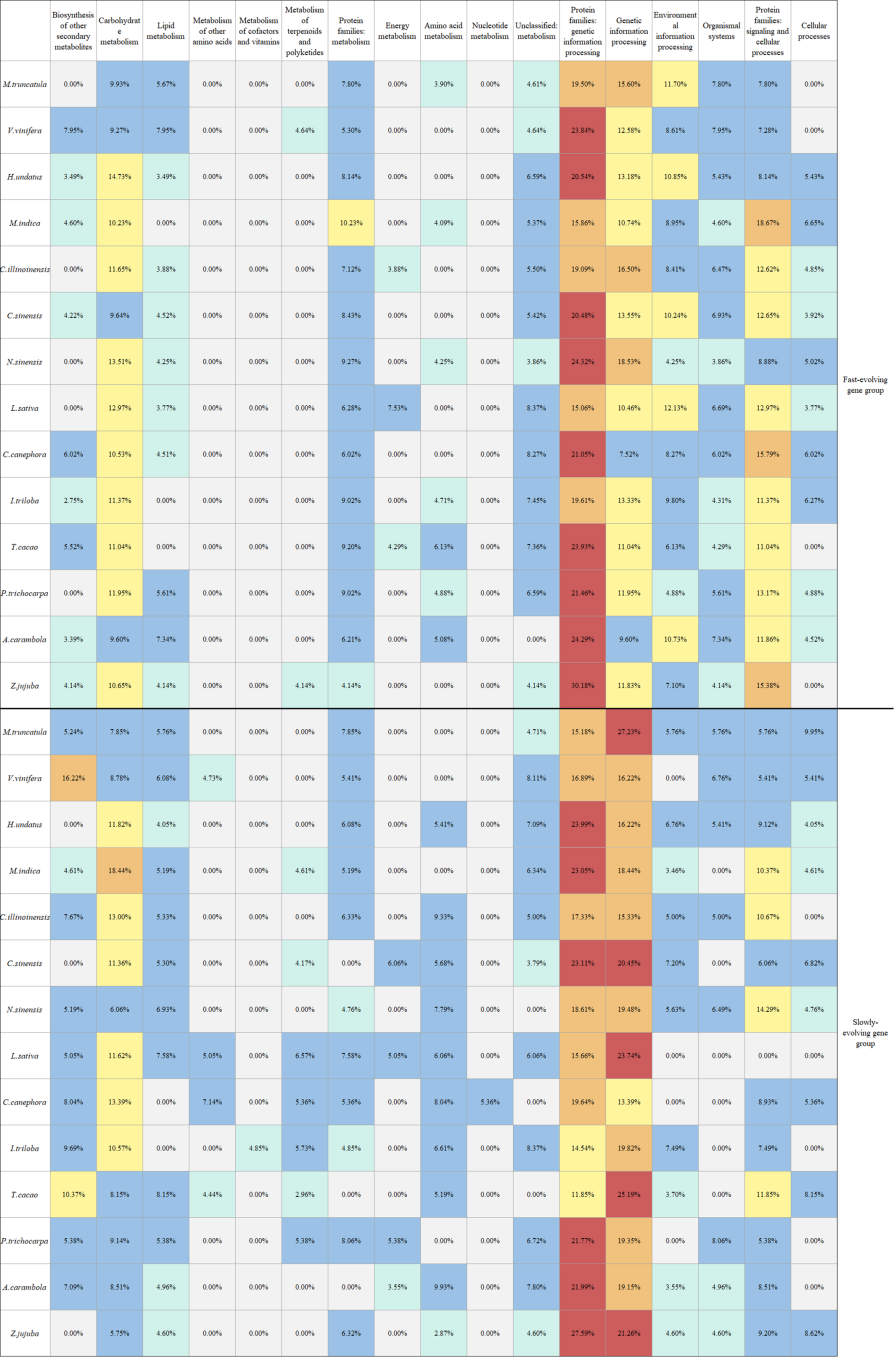
Distribution of biological pathway in the different evolving gene groups. The horizontal axis presents various biological pathways, while the vertical axis corresponds to different species. Among them, the differences in colors intuitively reflect the proportional relationship of genes participating in specific biological pathways within the corresponding species.

Often, the largest proportion of genes in both groups were involved in pathways related to “Genetic information processing” or “Protein families of genetic information processing.” An only exception was found with *M. indica*, in which the largest proportion of the fast-evolving genes were involved in pathways relating to “Protein families of signaling and cellular processes.” Interestingly, we found that the fast-evolving genes in all species and the slowly evolving ones in 13 species were not involved in the pathways relating to “Metabolism of cofactors and vitamins” (the exceptional one: *I. triloba*) or “Nucleotide metabolism” (the exceptional one: *C. canephora*). Similarly, the fast-evolving genes were not involved in pathways relating to “Metabolism of other amino acids” in all selected species, while the slowly evolving genes in four species were involved to an unneglectable percentage (4.44%–7.14%) (**Figure 6**).

**Figure 7:**
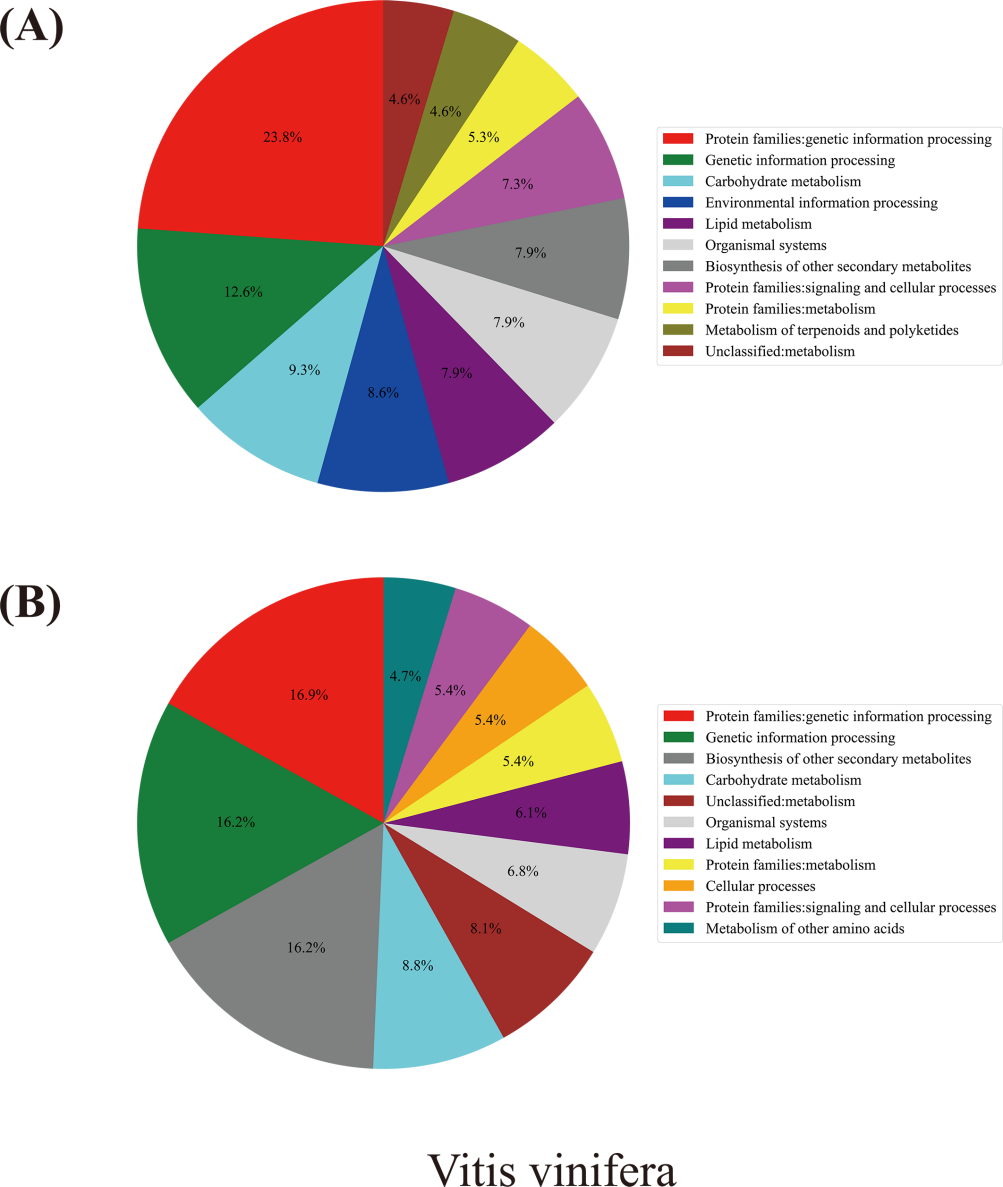
KEGG analysis results for grapevines. **(A)** Grapevine fast-evolving gene group pathway enrichment results. **(B)** Grapevine slowly evolving gene group pathway enrichment results.

Taking grapevine as an example (**Figure 7**), according to the annotation analysis of the Kyoto Encyclopedia of Genes and Genomes (KEGG), a Chi-square test showed that genes evolving at different rates were significantly divergently related to biological pathways (χ² = 43.976, p-value = 0.0001) (**Supplementary Table S6**). Actually, for the fast-evolving group in grapevines, genes involved in the “metabolism” category accounted for ~40% of the total, the most of which were divided into two groups: “carbohydrate metabolism” and “lipid metabolism.” Approximately 36% of the fast-evolving were related to “genetic information processing” and 9% to “environmental information processing.” As to the slowly evolving group, 50% of them belonged to the “metabolism” category, 10% more than those in the fast-evolving group. Among the “metabolism” category, the slowly evolving genes were mainly related to “biosynthesis of other secondary metabolites” (16%) and “carbohydrate metabolism” (9%), while genes related to “genetic information processing,” “cellular processes,” and “organismal systems” accounted for 33%, 10%, and 7%, respectively.

## Discussion

Polyploidy events or WGD have played a significant role in the evolutionary history of dicotyledons (Otto and Whitton, 2000; Soltis et al., 2009). Researches in the past 20 years revealed that all angiosperms and possibly all seed plants have a polyploid ancestor. Genomic comparisons based on sequenced genomes indicate that angiosperm plants have been affected by one or more episodes of polyploidization (Soltis). Approximately 50 polyploidization events have thus far been accurately identified across plant phylogenetic trees through genome sequencing and comparative genomic analysis (Vanneste et al., 2014; Cheng et al., 2018; Ren et al., 2018).

Grapevine is often used for investigating the WGD events of eudicot genomes since its genome underwent minimal DNA rearrangements following the ceWGT (Lodhi and Reisch, 1995; Velasco et al., 2007). Here, the model status of grapevine gains a new dimension of support that it has evolved more slowly than many other species studied to date. The grapevine genome is by far the closest to the ancestral genome of eudicot common ancestor (Jaillon et al., 2007). In contrast, most other species have experienced widespread genomic re-patterning resulting in chromosome fusions and, therefore, reduction in chromosome numbers. In summary, grapevine has distinct advantages among the known eudicots, so far, making it a good reference to clarify evolutionary variations, often resulting from additional polyploidization, occurring in other eudicot genomes.

The simultaneous duplication of genes provided by the common hexaploidization event (ceWGT) offers a unique research opportunity. Fortunately, the preservation of thousands of genes across various plant genomes not only enriches the duplicated gene pool to explore their genetic innovation during eudicot evolution but also enables reasonable inferences about the divergent evolutionary rates and paces of different plants. Furthermore, since plants can evolve at much divergent paces due to origination and distribution in different locations on the Earth, the existence of simultaneously duplicated genes produced in their common ancestor allows us to correct Ks values at the whole-genome scale and reasonably re-estimate key events during their respective evolution, such as polyploidization and speciation.

Therefore, by reviewing relevant literature, we collected the estimated times of relevant evolutionary events from previous studies and compared them with the dates obtained from the present research. A genomic synteny analysis based on the self-comparison of mango coding genes strongly supports a recent WGD event approximately 20 to 40 million years ago by mapping the WGD event onto the phylogeny. Here, we narrowed this time frame to a more precise range from 22 to 25 million years ago (Wang et al., 2020b). Two ancient WGD events were inferred in shea’s evolutionary past, one prior to the Astrid–Rosid divergence (116–126 Mya) and the other at the root of the order Ericales (65–90 Mya) (Xia et al., 2017; Wu et al., 2019; Yang et al., 2020a; Hale et al., 2021). Our study narrowed down the time estimates for the recent WGD event to approximately 50 to 56 million years ago. An analysis of gene collinearity has demonstrated that the tea plant’s genome has undergone two rounds of whole-genome duplications (WGDs) estimated to have taken place approximately 30 to 40 million years ago and approximately 90 to 100 million years ago (Wei et al., 2018). However, our findings suggest a slightly different timeline, with these duplication events occurring approximately 41 to 46 million years ago and 115 to 130 million years ago.

In the present study, the analysis of 28 eudicot plants revealed a striking variation in evolutionary rates, with differences of up to 68.04%. This variation is particularly pronounced among species that have experienced additional polyploidization events beyond the common hexaploidization event (ceWGT). We observed a clear correlation between the number of polyploidy events and Ks values indicating that each round of polyploidization introduces new genetic diversity, thereby accelerating the pace of evolution. This may have been caused by at least two reasons. As to the classical evolutionary theory, the existence of duplicated genes could buffer the mutation in one copy or both copies of the duplicated genes produced by the WGD (Yan et al., 2024). One duplicated gene might preserve the main function of their ancestral gene before the WGD, while the other might evolve new function referred to as neofunctionalization. There is also a possibility that the two duplicated genes split the ancestral gene’s function resulting in a phenomenon of subfunctionalization. As to previous reports, a more complex combination of subdivision of ancestral gene’s function might exist (Rastogi and Liberles, 2005). Notably, the elevation in gene evolutionary rates could result from illegitimate recombination between duplicated genes each residing on homeologous chromosomes produced by the WGD. A comparison of grass genomes revealed clear evidence of the occurrence of homeologous recombination, which should have been frequent during the early days after the WGD, and may have lasted for tens of million years between the terminal regions of homeologous chromosomes (Wang et al., 2009, 2011b).

Furthermore, the differences in evolutionary rates have led to significant variations in Ks values among genes, which in turn affect the involvement of genes in metabolic pathways. Genes that evolve rapidly, with higher Ks values, tend to play diverse roles in metabolic pathways, which may be related to their rapid response to environmental pressures and niche differentiation. The rapid evolution of these genes may endow them with new or improved biological functions, thereby enhancing the plant’s ability to adapt to new environments.

In summary, polyploidization events have significantly driven plant evolution by introducing new genetic diversity and accelerating the evolutionary rates of genes (Van De Peer et al., 2017). These changes in rates not only affect the variation and retention of genes but also their participation and function in metabolic pathways, thus playing a key role in shaping plant adaptability and the metabolic network. These findings emphasize the important role of polyploidization in the evolutionary process of plants and provide new insights into how the evolutionary rates of genes affect plant metabolic functions and ecological adaptability.

## Materials and methods

### Plant genome data materials

We collected 28 high-quality, chromosome-level core eudicot plant genomes (mainly from NCBI and PHYTOZOME). The data materials mainly include genome annotation files (General feature format, GFF), gene translated protein files (Peptide, PEP), and coding sequence files (CDS) (**Supplementary Table S1**). Python scripts (https://github.com/SunPengChuan/wgdi) were used to process data format to facilitate subsequent research.

### Multiple sequence alignment and inference of gene collinearity

The first step is to perform multiple sequence alignment. According to the sequence alignment tool BLAST (Altschul et al., 1990), select the -blastp module to perform homology alignment within and between genomes of the selected species’ genomic protein sequences. The E-value threshold of the output result is set to 1e^−5^ to accommodate the duplicated genes produced by paleopolyploidization ∼10 Mya, and the output file format (-outfmt) is set to 6 during the specific operation.

Based on the previously obtained homologous sequence alignment result file (Blast file), combined with the genome annotation file (Gff file) and chromosome length file (Lens file), we used the -d module implemented in WGDI (Sun et al., 2022) to draw homologous gene dot plots. The dots of different colors (red, blue, and gray) in the dot plots represent the level of similarity of their gene pairs. According to the dot plots of homologous genes, homologous collinearity was inferred within each genome and between genomes. Next, we used the -icl module to perform collinearity analysis and obtained the collinearity regions, described by scores, statistical significance, collinear gene numbers, etc.

### Calculation of synonymous nucleotide substitutions

According to the collinear genes obtained previously, the Ks values were calculated by combining the cds and pep files. Here, the -Ks module in WGDI was used. This module used Muscle software (Rc, 2021) to perform protein binding based on the protein sequence, used pal2pal.pl to convert the protein binding into codon binding based on the CDS sequence, and finally calculated Ks using yn00 from PAML (Yang, 2007).

### Synonymous substitution correction

We performed the Ks correction among species by aligning the ceWGD peaks of Ks values between duplicated genes in each genome (**Figure 5A**) to the peak location in grapevine.

Using the aforementioned method for calculating Ks values, we calculated the correction coefficients for duplicated genes within individual genomes. We used $Ks^{'}$ to denote the Ks values after correction, which was obtained by multiplying the original Ks values by the coefficient (Zhuang et al., 2019; Song et al., 2021b).

For any species q, its correction coefficient is defined as q, its correction coefficient is defined as $C_{q}=Ks^{'}/Ks=P_{Vi}/P_{q}=k$ ; thus, $Ks_{q}^{'}=Ks\times \;C_{q}=Ks\times \;\left(P_{Vi}/P_{q}\right)=Ks\times k$ . Among them, $P_{q}$ represents the peak value of the Ks distribution of species q.

For example, the correction coefficient of duplicated genes in diploid wheat *C. sinensis* was defined as $C_{Ca}=Ks^{'}/Ks=P_{Vi}/P_{Ca}=1.0608$ ; therefore, $Ks^{'}=Ks\times \;C_{Ca}=Ks\times \;\left(P_{Vi}/P_{Ca}\right)=Ks\times 1.0608$ .

## Data Availability

The original contributions presented in the study are included in the article/supplementary material. Further inquiries can be directed to the corresponding author.

## References

[B1] Altschul S. F. Gish W. Miller W. Myers E. W. Lipman D. J. (1990). Basic local alignment search tool. J. Mol. Biol. 215, 403–410. doi: 10.1006/jmbi.1990.9999 2231712

[B2] Barker M. S. Husband B. C. Pires J. C. (2016). Spreading Winge and flying high: The evolutionary importance of polyploidy after a century of study. Am. J. Bot. 103, 1139–1145. doi: 10.3732/ajb.1600272 27480249

[B3] Barker M. S. Vogel H. Schranz M. E. (2009). Paleopolyploidy in the Brassicales: Analyses of the*Cleome* Transcriptome Elucidate the History of Genome Duplications in*Arabidopsis* and Other Brassicales. Genome Biol. Evol. 1, 391–399. doi: 10.1093/gbe/evp040 20333207 PMC2817432

[B4] Bowers J. E. Chapman B. A. Rong J. Paterson A. H. (2003). Unravelling angiosperm genome evolution by phylogenetic analysis of chromosomal duplication events. Nature 422, 433–438. doi: 10.1038/nature01521 12660784

[B5] Cheng F. Wu J. Cai X. Liang J. Freeling M. Wang X. (2018). Gene retention, fractionation and subgenome differences in polyploid plants. Nat. Plants 4, 258–268. doi: 10.1038/s41477-018-0136-7 29725103

[B6] Clark L. V. Jin X. L. Petersen K. K. Anzoua K. G. Bagmet L. Chebukin P.. (2019). Population structure of*Miscanthus sacchariflorus* reveals two major polyploidization events, tetraploid-mediated unidirectional introgression from diploid*M. sinensis*, and diversity centred around the Yellow Sea. Ann. Bot. 124, 731–748. doi: 10.1093/aob/mcy161 30247525 PMC6821896

[B7] Doyle J. J. Coate J. E. (2019). Polyploidy, the nucleotype, and novelty: the impact of genome doubling on the biology of the cell. Int. J. Plant Sci. 180, 1–52. doi: 10.1086/700636

[B8] Fang C. Yang M. Y. Tang Y. C. Zhang L. Zhao H. A. Ni H. J.. (2023). Dynamics of cis- regulatory sequences and transcriptional divergence of duplicated genes in soybean. Proc. Natl. Acad. Sci. United States America 120, 11. doi: 10.1073/pnas.2303836120 PMC10622917 37871213

[B9] Gaeta R. T. Chris Pires J. (2010). Homoeologous recombination in allopolyploids: the polyploid ratchet. New Phytol. 186, 18–28. doi: 10.1111/j.1469-8137.2009.03089.x 20002315

[B10] Hale I. Ma X. Melo A. T. O. Padi F. K. Hendre P. S. Kingan S. B.. (2021). Genomic resources to guide improvement of the shea tree. Front. Plant Sci. 12. doi: 10.3389/fpls.2021.720670 PMC8459026 34567033

[B11] Hu G. J. Grover C. E. Vera D. L. Lung P. Y. Girimurugan S. B. Miller E. R.. (2024). Evolutionary dynamics of chromatin structure and duplicate gene expression in diploid and allopolyploid cotton. Mol. Biol. Evol. 41, 28. doi: 10.1093/molbev/msae095 PMC11140268 38758089

[B12] Jaillon O. Aury J. M. Noel B. Policriti A. Clepet C. Casagrande A.. (2007). The grapevine genome sequence suggests ancestral hexaploidization in major angiosperm phyla. Nature 449, 463–U465. doi: 10.1038/nature06148 17721507

[B13] Jiao Y. N. Leebens-Mack J. Ayyampalayam S. Bowers J. E. McKain M. R. McNeal J.. (2012). A genome triplication associated with early diversification of the core eudicots. Genome Biol. 13, 14. doi: 10.1186/gb-2012-13-1-r3 PMC3334584 22280555

[B14] Jiao Y. Wickett N. J. Ayyampalayam S. Chanderbali A. S. Landherr L. Ralph P. E.. (2011). Ancestral polyploidy in seed plants and angiosperms. Nature 473, 97–100. doi: 10.1038/nature09916 21478875

[B15] Kaul S. Koo H. L. Jenkins J. Rizzo M. Rooney T. Tallon L. J.. (2000). Analysis of the genome sequence of the flowering plant*Arabidopsis thaliana* . Nature 408, 796–815. doi: 10.1038/35048692 11130711

[B16] Li Z. Defoort J. Tasdighian S. Maere S. Van de Peer Y. De Smet R. (2016). Gene duplicability of core genes is highly consistent across all angiosperms. Plant Cell 28, 326–344. doi: 10.1105/tpc.15.00877 26744215 PMC4790876

[B17] Liu Y. Z. Wang J. P. Ge W. N. Wang Z. Y. Li Y. X. Yang N. S.. (2017). Two highly similar poplar paleo-subgenomes suggest an autotetraploid ancestor of salicaceae plants. Front. Plant Sci. 8. doi: 10.3389/fpls.2017.00571 PMC5388744 28446920

[B18] Lodhi M. A. Reisch B. I. (1995). Nuclear DNA content of vitis species, cultivars, and other genera of the vitaceae. Theor. Appl. Genet. 90, 11–16. doi: 10.1007/bf00220990 24173778

[B19] Madlung A. (2013). Polyploidy and its effect on evolutionary success: old questions revisited with new tools. Heredity 110, 99–104. doi: 10.1038/hdy.2012.79 23149459 PMC3554449

[B20] Murat F. Armero A. Pont C. Klopp C. Salse J. (2017). Reconstructing the genome of the most recent common ancestor of flowering plants. Nat. Genet. 49, 490–49+. doi: 10.1038/ng.3813 28288112

[B21] Otto S. P. Whitton J. (2000). Polyploid incidence and evolution. Annu. Rev. Genet. 34, 401–437. doi: 10.1146/annurev.genet.34.1.401 11092833

[B22] Paterson A. H. Bowers J. E. Chapman B. A. (2004). Ancient polyploidization predating divergence of the cereals, and its consequences for comparative genomics. Proc. Natl. Acad. Sci. U. S. A. 120, 9903–9908. doi: 10.1073/pnas.0307901101 PMC470771 15161969

[B23] Paterson A. H. Wendel J. F. Gundlach H. Guo H. Jenkins J. Jin D.. (2012). Repeated polyploidization of Gossypium genomes and the evolution of spinnable cotton fibres. Nature 492, 423–427. doi: 10.1038/nature11798 23257886

[B24] Rastogi S. Liberles D. A. (2005). Subfunctionalization of duplicated genes as a transition state to neofunctionalization. BMC Evolutionary Biol. 5, 7. doi: 10.1186/1471-2148-5-28 PMC1112588 15831095

[B25] Rc E.J.C.S.H.L. (2021). MUSCLE v5 enables improved estimates of phylogenetic tree confidence by ensemble bootstrapping. Cold Spring Harbor Lab. 2021. doi: 10.1101/2021.06.20.449169

[B26] Ren R. Wang H. F. Guo C. C. Zhang N. Zeng L. P. Chen Y. M.. (2018). Widespread whole genome duplications contribute to genome complexity and species diversity in angiosperms. Mol. Plant 11, 414–428. doi: 10.1016/j.molp.2018.01.002 29317285

[B27] Schmutz J. Cannon S. B. Schlueter J. Ma J. X. Mitros T. Nelson W.. (2010). Genome sequence of the palaeopolyploid soybean. Nature 463, 178–183. doi: 10.1038/nature08670 20075913

[B28] Shen S. Li Y. Wang J. Wei C. Wang Z. Ge W.. (2021). Illegitimate recombination between duplicated genes generated from recursive polyploidizations accelerated the divergence of the genus arachis. Genes (Basel) 12 (12). doi: 10.3390/genes12121944 PMC8701993 34946893

[B29] Soltis D. E. Albert V. A. Leebens-Mack J. Bell C. D. Paterson A. H. Zheng C. F.. (2009). POLYPLOIDY AND ANGIOSPERM DIVERSIFICATION. Am. J. Bot. 96, 336–348. doi: 10.3732/ajb.0800079 21628192

[B30] Soltis P. S. Marchant D. B. Van de Peer Y. Soltis D. E. (2015). Polyploidy and genome evolution in plants. Curr. Opin. Genet. Dev. 35, 119–125. doi: 10.1016/j.gde.2015.11.003 26656231

[B31] Soltis P. S. Soltis D. E. (2016). Ancient WGD events as drivers of key innovations in angiosperms. Curr. Opin. Plant Biol. 30, 159–165. doi: 10.1016/j.pbi.2016.03.015 27064530

[B32] Song X. M. Sun P. C. Yuan J. Q. Gong K. Li N. Meng F. B.. (2021a). The celery genome sequence reveals sequential paleo-polyploidizations, karyotype evolution and resistance gene reduction in apiales. Plant Biotechnol. J. 19, 731–744. doi: 10.1111/pbi.13499 33095976 PMC8051603

[B33] Song X. M. Wei Y. P. Xiao D. Gong K. Sun P. C. Ren Y. M.. (2021b). *Brassica carinata* genome characterization clarifies U's triangle model of evolution and polyploidy in*Brassica* . Plant Physiol. 186, 388–406. doi: 10.1093/plphys/kiab048 33599732 PMC8154070

[B34] Sun P. C. Jiao B. B. Yang Y. Z. Shan L. X. Li T. Li X. N.. (2022). WGDI: A user-friendly toolkit for evolutionary analyses of whole-genome duplications and ancestral karyotypes. Mol. Plant 15, 1841–1851. doi: 10.1016/j.molp.2022.10.018 36307977

[B35] Tuskan G. A. DiFazio S. Jansson S. Bohlmann J. Grigoriev I. Hellsten U.. (2006). The genome of black cottonwood,*Populus trichocarpa* (Torr. & Gray). Science 313, 1596–1604. doi: 10.1126/science.1128691 16973872

[B36] Van De Peer Y. Mizrachi E. Marchal K. (2017). The evolutionary significance of polyploidy. Nat. Rev. Genet. 18, 411–424. doi: 10.1038/nrg.2017.26 28502977

[B37] Vanneste K. Baele G. Maere S. Van de Peer Y. (2014). Analysis of 41 plant genomes supports a wave of successful genome duplications in association with the Cretaceous-Paleogene boundary. Genome Res. 24, 1334–1347. doi: 10.1101/gr.168997.113 24835588 PMC4120086

[B38] Vanneste K. Van de Peer Y. Maere S. (2013). Inference of genome duplications from age distributions revisited. Mol. Biol. Evol. 30, 177–190. doi: 10.1093/molbev/mss214 22936721

[B39] Velasco R. Zharkikh A. Troggio M. Cartwright D. A. Cestaro A. Pruss D.. (2007). A high quality draft consensus sequence of the genome of a heterozygous grapevine variety. PloS One 2, 18. doi: 10.1371/journal.pone.0001326 PMC2147077 18094749

[B40] Vision T. J. Brown D. G. Tanksley S. D. (2000). The origins of genomic duplications in*Arabidopsis* . Science 290, 2114–2117. doi: 10.1126/science.290.5499.2114 11118139

[B41] Wang X. Y. Gowik U. Tang H. B. Bowers J. E. Westhoff P. Paterson A. H. (2009). Comparative genomic analysis of C4 photosynthetic pathway evolution in grasses. Genome Biol. 10, 58. doi: 10.1186/gb-2009-10-6-r68 PMC2718502 19549309

[B42] Wang X. Y. Guo H. Wang J. P. Lei T. Y. Liu T. Wang Z. Y.. (2016). Comparative genomic de-convolution of the cotton genome revealed a decaploid ancestor and widespread chromosomal fractionation. New Phytol. 209, 1252–1263. doi: 10.1111/nph.13689 26756535

[B43] Wang P. Luo Y. F. Huang J. F. Gao S. H. Zhu G. P. Dang Z. G.. (2020b). The genome evolution and domestication of tropical fruit mango. Genome Biol. 21, 17. doi: 10.1186/s13059-020-01959-8 32143734 PMC7059373

[B44] Wang J. P. Qin J. Sun P. C. Ma X. L. Yu J. G. Li Y. X.. (2019). Polyploidy index and its implications for the evolution of polyploids. Front. Genet. 10. doi: 10.3389/fgene.2019.00807 PMC6746930 31552101

[B45] Wang J. L. Song B. W. Yang M. R. Hu F. B. Qi H. L. Zhang H. Z.. (2024). Deciphering recursive polyploidization in Lamiales and reconstructing their chromosome evolutionary trajectories. Plant Physiol. 195, 2143–2157. doi: 10.1093/plphys/kiae151 38482951

[B46] Wang J. P. Sun P. C. Li Y. X. Liu Y. Z. Yang N. S. Yu J. G.. (2018). An overlooked paleotetraploidization in cucurbitaceae. Mol. Biol. Evol. 35, 16–26. doi: 10.1093/molbev/msx242 29029269 PMC5850751

[B47] Wang X. Y. Tang H. B. Paterson A. H. (2011b). Seventy million years of concerted evolution of a homoeologous chromosome pair, in parallel, in major poaceae lineages. Plant Cell 23, 27–37. doi: 10.1105/tpc.110.080622 21266659 PMC3051248

[B48] Wang X. Y. Wang J. P. Jin D. C. Guo H. Lee T. H. Liu T.. (2015). Genome alignment spanning major poaceae lineages reveals heterogeneous evolutionary rates and alters inferred dates for key evolutionary events. Mol. Plant 8, 885–898. doi: 10.1016/j.molp.2015.04.004 25896453

[B49] Wang X. W. Wang H. Z. Wang J. Sun R. F. Wu J. Liu S. Y.. (2011a). The genome of the mesopolyploid crop species*Brassica rapa* . Nat. Genet. 43, 1035–U1157. doi: 10.1038/ng.919 21873998

[B50] Wang J. P. Yu J. G. Sun P. C. Li C. Song X. M. Lei T. Y.. (2020a). Paleo-polyploidization in lycophytes. Genomics Proteomics Bioinf. 18, 333–340. doi: 10.1016/j.gpb.2020.10.002 PMC7801247 33157303

[B51] Wei C. L. Yang H. Wang S. B. Zhao J. Liu C. Gao L. P.. (2018). Draft genome sequence of*Camellia sinensis* var.*sinensis* provides insights into the evolution of the tea genome and tea quality. Proc. Natl. Acad. Sci. United States America 115, E4151–E4158. doi: 10.1073/pnas.1719622115 PMC5939082 29678829

[B52] Wu H. L. Ma T. Kang M. H. Ai F. D. Zhang J. L. Dong G. Y.. (2019). A high-quality*Actinidia chinensis* (kiwifruit) genome. Horticulture Res. 6, 9. doi: 10.1038/s41438-019-0202-y PMC6804796 31645971

[B53] Xia E. H. Zhang H. B. Sheng J. Li K. Zhang Q. J. Kim C.. (2017). The tea tree genome provides insights into tea flavor and independent evolution of caffeine biosynthesis. Mol. Plant 10, 866–877. doi: 10.1016/j.molp.2017.04.002 28473262

[B54] Xiao L. Q. Li Q. Q. (2017). Phylogeography and allopolyploidization of*Magnolia* sect.*Gynopodium* (Magnoliaceae) in subtropical China. Plant Systematics Evol. 303, 957–967. doi: 10.1007/s00606-017-1409-8

[B55] Yan X. Shi G. Sun M. Shan S. Chen R. Li R.. (2024). Genome evolution of the ancient hexaploid Platanus × acerifolia (London planetree). Proc. Natl. Acad. Sci. U.S.A. 121, e2319679121. doi: 10.1073/pnas.2319679121 38830106 PMC11181145

[B56] Yang Z. H. (2007). PAML 4: Phylogenetic analysis by maximum likelihood. Mol. Biol. Evol. 24, 1586–1591. doi: 10.1093/molbev/msm088 17483113

[B57] Yang F. S. Nie S. Liu H. Shi T. L. Tian X. C. Zhou S. S.. (2020a). Chromosome-level genome assembly of a parent species of widely cultivated azaleas. Nat. Commun. 11, 13. doi: 10.1038/s41467-020-18771-4 33077749 PMC7572368

[B58] Yang Y. Z. Sun P. C. Lv L. K. Wang D. L. Ru D. F. Li Y.. (2020b). Prickly waterlily and rigid hornwort genomes shed light on early angiosperm evolution. Nat. Plants 6, 215–222. doi: 10.1038/s41477-020-0594-6 32094642 PMC8075997

[B59] You J. Q. Liu Z. P. Qi Z. Y. Ma Y. Z. Sun M. L. Su L.. (2023). Regulatory controls of duplicated gene expression during fiber development in allotetraploid cotton. Nat. Genet. 55, 1987–198+. doi: 10.1038/s41588-023-01530-8 37845354 PMC10632151

[B60] Zhuang W. J. Chen H. Yang M. Wang J. P. Pandey M. K. Zhang C.. (2019). The genome of cultivated peanut provides insight into legume karyotypes, polyploid evolution and crop domestication. Nat. Genet. 51, 865–86+. doi: 10.1038/s41588-019-0402-2 31043757 PMC7188672

